# ACCURACY OF ELASTOGRAPHY IN THE ASSESSMENT OF REDUCTION IN LIVER STEATOSIS AND FIBROSIS IN THE EARLY POSTOPERATIVE PERIOD AFTER BARIATRIC SURGERY

**DOI:** 10.1590/0102-672020220002e1671

**Published:** 2022-08-26

**Authors:** Ramon Rawache LIMA, José Huygens Parente GARCIA, Marina Seixas STUDART, Fernando Siqueira PINHEIRO, João Odilo Gonçalves PINTO, Leonardo Adolpho SALES, Lucas Marrocos SOARES, Priscilla de Almeida SANTOS

**Affiliations:** 1 Universidade Federal do Ceará, Department of Surgery – Fortaleza (CE), Brazil;; 2 Walter Cantídeo University Hospital, Liver Transplant Unit – Fortaleza (CE), Brazil;; 3 Centro Universitário Christus, Surgery Department – Fortaleza (CE), Brazil;; 4 Walter Cantídeo University Hospital, Digestive System Surgery Unit – Fortaleza (CE), Brazil;; 5 Fortaleza General Hospital, Liver Transplant Unit – Fortaleza (CE), Brazil.

**Keywords:** Non-alcoholic Fatty Liver Disease, Bariatric Surgery, Liver Cirrhosis, Hepatopatia Gordurosa não Alcoólica, Cirurgia Bariátrica, Cirrose Hepática

## Abstract

**BACKGROUND:**

Nonalcoholic hepatic steatosis is found in most obese patients and has a strong association with metabolic syndrome. The Roux-en-Y gastric bypass and the sleeve gastrectomy are the two techniques of bariatric surgery. Patients who underwent bariatric surgery have regression of nonalcoholic steatohepatitis due to a reduction in body mass index and changes in incretin hormones.

**AIMS:**

This study aimed to analyze the acuity of elastography in the regression of hepatic steatosis and fibrosis in obese patients undergoing Roux-en-Y gastric bypass and sleeve gastrectomy 2 months after surgery.

**METHODS:**

Patients in the preoperative period of bariatric surgery underwent an anthropometric evaluation and hepatic elastography to quantify fibrosis and hepatic steatosis. Two months after surgery, the same evaluation was performed again.

**RESULTS:**

All 17 patients who met the inclusion criteria participated in the study. Out of this, nine underwent sleeve gastrectomy, and eight underwent Roux-en-Y gastric bypass. The Roux-en-Y gastric bypass group had lower fibrosis levels postoperatively compared to preoperatively (p=0.029, p<0.05). As for steatosis, patients who underwent Roux-en-Y gastric bypass had lower postoperative values (p=0.01, p<0.05). There was also a reduction in fibrosis postoperatively in the sleeve gastrectomy group compared to preoperatively (p=0.037, p<0.05).

**CONCLUSIONS:**

Elastography accurately demonstrated decreased hepatic steatosis and fibrosis in the early postoperative period of bariatric surgery. Moreover, Roux-en-Y gastric bypass and sleeve gastrectomy are suitable surgical methods to improve hepatic steatosis and fibrosis within 2 months postoperatively.

## RESUMO

**RACIONAL:**

A esteatose hepática não alcoólica é encontrada na maioria dos pacientes com obesidade e tem forte associação com a síndrome metabólica. O *bypass* gástrico em Y de Roux e a gastrectomia vertical são as duas técnicas de cirurgia bariátrica. Pacientes que são submetidos a cirurgia bariátrica tem regressão da esteatose hepática não alcoólica devido a redução do índice de massa corpórea e mudanças dos hormônios incretínicos.

**OBJETIVOS:**

analisar a acuidade da elastografia na regressão da esteatose e fibrose hepáticas em pacientes obesos submetidos a *bypass* gástrico em Y de Roux e gastrectomia vertical dois meses após a cirurgia.

**MÉTODOS:**

Pacientes em pré-operatório de cirurgia bariátrica foram submetidos a avaliação antropométrica e elastografia hepática para quantificação de fibrose e esteatose hepática. Dois meses após a cirurgia, a mesma avaliação foi realizada novamente.

**RESULTADOS:**

Dezessete pacientes preencheram todos os critérios de inclusão no estudo. Nove foram submetidos a gastrectomia vertical e 8 a *bypass* gástrico em Y de Roux. O grupo *bypass* gástrico em Y de Roux apresentou níveis de fibrose mais baixos no pós-operatório em comparação com o pré-operatório, (p=0,029, p<0,05). Quanto à esteatose, os pacientes que realizaram *bypass* gástrico em Y de Roux apresentaram valores menores no pós-operatório (p=0,01, p<0,05). No grupo gastrectomia vertical, também houve redução da fibrose no pós-operatório em relação ao pré-operatório (p=0,037, p<0,05).

**CONCLUSÕES:**

A elastografia mostrou acuidade para demonstrar diminuição da esteatose e fibrose hepáticas, no pós-operatório precoce de cirurgia bariátrica. Além disso, *bypass* gástrico em Y de Roux e gastrectomia vertical são métodos cirúrgicos adequados para melhorar a esteatose e fibrose hepática em um período de 2 meses de pós-operatório.

## INTRODUCTION

Primary obesity, determined by the imbalance between energy intake and expenditure, is a major public health problem worldwide. There are an estimated 2.1 billion obese or overweight people across the world, with 2.5 million obesity-related deaths per year^16^.

There are numerous clinical complications of obesity that impact morbidity and mortality, such as acute myocardial infarction or stroke. Among these complications, nonalcoholic hepatic steatosis (NASH) is one of the most frequent in about 95% of obese patients who undergo bariatric surgery^23^.

In recent years, NASH has had significant clinical importance and is strongly related to metabolic syndrome^3^. NASH can progress to hepatic fibrosis. One-third of patients with NASH will progress to hepatic fibrosis^5^, indicating that NASH is one of the most critical current indications for liver transplantation^24^.

Liver cirrhosis and hepatocellular carcinoma (HCC) are the leading causes of liver-related death in patients with NASH^8^. HCC can even appear in patients without cirrhosis. The indication for liver transplantation due to HCC is 3.64 times higher in patients with NASH and a body mass index (BMI) >30^4^.

Some authors advocated bariatric surgery prior to liver transplantation in patients with cirrhosis due to NASH^7^.

The two most commonly performed bariatric surgeries globally are sleeve gastrectomy (SG) and Roux-en-Y gastric bypass (RYGB). The SG consists of performing a vertical gastrectomy, in order to reduce the stomach volume, and is, therefore, a surgery classified as restrictive. The gastric bypass consists of confectioning a small gastric reservoir, excluding the rest of the stomach, and reconstructing the Roux-en-Y intestinal transit. Therefore, this procedure is restrictive and disabsortive^20^.

The indication criteria for these surgical modalities are well established, although there is nothing defined concerning NASH, despite some studies^2^.

The gold-standard procedure for diagnosing steatosis and hepatic fibrosis is liver biopsy, but it is a restricted procedure since it is an invasive method. Hence, the need for reliable noninvasive methods has propelled the emergence of some studies in this direction. There are reports of ordinary ultrasound, magnetic resonance imaging, and even laboratory tests and evaluation scores^25^.

Liver elastography has been a promising noninvasive method, and several previous studies have shown it to be a reliable and accurate test to assess hepatic steatosis and fibrosis^9,10^.

Therefore, the objective of this research was to quantify hepatic fibrosis and steatosis using elastography with the Fibroscan^®^ device, preoperatively, and then in the early postoperative period, in obese patients with indication for bariatric surgery. Patients selected for bariatric surgery were interviewed and invited to participate in the study. A new evaluation of the same parameters was performed in the second postoperative month — a hepatologist with adequate training to perform the examination and expertise in operating the Fibroscan^®^ performed elastography.

## METHODS

At Walter Cantídeo University Hospital (HUWC), patients are selected and prepared for surgical treatment under current legislation. Therefore, they all have primary obesity, are over 18 years old, and have a BMI over 40 or between 35 and 39.9 with comorbidities associated with obesity. In addition, patients undergo a multiprofessional evaluation, including routine consultations with a nutritionist, psychologist, and endocrinologist.

The choice of bariatric procedure indicated for each patient was based on relative criteria such as the concomitant presence of metabolic syndrome, gastroesophageal reflux disease, overweight, and others. The patient’s wishes were also considered, although not a preponderant factor. In the Digestive Surgery Service of HUWC, hepatic steatosis is not considered a criterion for the choice of surgical technique.

Exclusion criteria included the presence of complications indicating surgical reintervention, a diagnosis of cancer on histopathological examination of the surgical specimen, and the presence of previous liver disease.

The study was approved by the HUWC Ethics Committee and the National Ethics Committee for conducting research under number 74840317.0.0000.5045.

All patients were operated on by the same team and with standardization of surgical technique. Patients undergoing SG had the residual stomach calibrated with a 32F Foucher probe, while patients undergoing RYGB had gastric pouch calibrated with a 32F Foucher probe and 100 cm biliopancreatic loop.

Data normality was assessed using the Kolmogorov-Smirnov and Shapiro-Wilk tests in the statistical analysis. The assumption of homogeneity of variance was evaluated using Levene’s test. Bootstrapping procedures (1000 resamples; 95% BCa CI) were performed to obtain greater reliability of the results, correct for deviations from the normality of the sample distribution and differences between group sizes, and present a 95%CI (p<0.05) for the differences between means.

## RESULTS

A total of 17 patients were included in the study, with 8 undergoing RYGB and 9 undergoing SG. Although there was no randomized choice, both groups had similar characteristics. As for the gender distribution, there were seven women and one man in the RYGB group and all women in the SG group. Hence, there was no statistical difference in any factors, such as age, gender, preoperative BMI, preoperative weight, fibrosis, or preoperative steatosis, demonstrating that the groups in the preoperative period were similar (Table 1).

**Table 1 -:** Characteristics of each group before surgery.

	Total	SG	RYGB	p-value
Age (years)	45 (40–49)	43.5 (39–45)	49 (45–51)	0.134
Height (m)	1.55 (1.5–1.6)	1.52 (1.48–1.6)	1.58 (1.52–1.6)	0.210
Preoperative weight (kg)	109.5 (93–124)	102.13 (93.85–123.83)	112.55 (93–138)	0.441
Preoperative BMI (kg/m^2^)	43.9 (40–48.71)	42.99 (40.6–48.88)	44.42 (39.1–48.71)	0.773
Preoperative abdominal waist (cm)	120 (112–132)	114.5 (107.5–122)	125 (120–135)	0.092

SG: sleeve gastrectomy; RYGB: Roux-en-Y gastric bypass; BMI: body mass index.

Liver fibrosis and steatosis were measured before the intervention (pre-test) and after the intervention (post-test) in patients who underwent SG and RYGB. The results showed that in the RYGB group, liver fibrosis levels were lower postoperatively, with a mean of 1.67, when compared to preoperatively, mean of 2.44 (p=0.029, p<0.05). Similarly, these findings were also observed in the SG group, where the mean value of hepatic fibrosis was lower postoperatively, equal to 1 when compared with preoperatively, that was 1.25 (p=0.037, p<0.05). As for hepatic steatosis, patients who underwent RYGB had lower values postoperatively, with a mean of 1.22 (p=0.01, p<0.05) (Table 2).

**Table 2 -:** Preoperative and postoperative fibrosis and steatosis results.

Variables	Surgery type	
	SG	RYGB
Preoperative fibrosis	1.25±0.46 (1)	2.44±1.42 (3)
Postoperative fibrosis	1±0 (1)	1.67±1 (1)
Bootstrapping sample difference pre-post (95%CI)	0.25 (0.13 to 0.63)	0.78 (0.33 to 1.22)
p-value	0.037	0.029
Preoperative steatosis	2.5±0.93 (3)	2.33±1 (3)
Postoperative steatosis	2.13±0.83 (2)	1.22±1.09 (1)
Bootstrapping sample difference pre-post (95%CI)	0.38 (-0.25 to 1)	1.11 (0.67 to 1.67)
p	0.258	0.01

SG: sleeve gastrectomy; RYGB: Roux-en-Y gastric bypass; 95%CI: Índice Confidencial.

An important fact to be reported is that there was no statistical difference between the weight loss between the two groups in the postoperative period, similarly to the BMI reduction between the two groups (p=0.923, p>0.05), suggesting that weight loss alone would not be the main factor for the reduction of hepatic steatosis in 2 months (Table 3).

**Table 3 -:** Comparison of preoperative and postoperative weight and BMI between two groups.

	Total	SG	RYGB	p-value
Preoperative weight (kg)	109.5 (93–124)	102.13 (93.85–123.83)	112.55 (93–138)	0.441
Postoperative weight (kg)	95 (81.85–109.3)	100.23 (83.02–109.15)	95 (77.38–111)	0.048
Preoperative BMI (kg/m²)	43.9 (40–48.71)	42.99 (40.6–48.88)	44.42 (39.1–48.71)	0.773
Postoperative BMI (kg/m²)	36.66 (35.01–43.7)	37.5 (34.93–44.47)	36.66 (35.01–41.11)	0.923

SG: sleeve gastrectomy; RYGB: Roux-en-Y gastric bypass; BMI: body mass index.

## DISCUSSION

Bariatric surgery is now considered the gold-standard treatment for grade II obesity-associated comorbidities and grade III obesity. Therefore, surgery can considerably reduce the risks of the complications of obesity and its comorbidities^11^.

Besides weight loss, bariatric surgery can also guarantee good results in controlling diabetes mellitus^6,12,13^, hypertension, dyslipidemia, and other parameters for metabolic syndrome^14,15^, showing that RYGB has better results when compared to SG^14,15,20^. Schauer et al.^21^ showed that surgical treatment, either RYGB or SG, leads to better glycemic control when compared with optimized clinical treatment for type 2 diabetes mellitus. RYGB shows better effectiveness in this glycemic control concerning both surgical techniques in this same study. Similar results were determined in a study by Pournaras et al.^19^.

Glycemic control can even be achieved before the expected total weight loss. There are bariatric surgery services that program the reduction of hypoglycemic medications and other medications for diseases involving metabolic syndrome, even in the early postoperative period^18^.

As for NAHS, a very prevalent complication among obese people, there are also criteria for cure after bariatric surgery. Lassailly et al.^13^ showed that 85% of patients undergoing bariatric surgery have regression of hepatic steatosis after 1 year of follow-up. Luo et al. showed that 83.7% of patients after bariatric surgery had regression of steatosis after 6 months, regardless of the surgical technique used^14^.

The reduction of hepatic steatosis in post-bariatric patients is related to direct weight loss, but there are also factors independent of weight loss. Incretins, such as GLP-1, GLP-2, and ghrelin, play an essential role in reducing hepatic steatosis. The anatomical changes promoted by surgery, especially the RYGB, lead to an increase in these substances^21^.

Nickel et al. showed that RYGB was more effective in reducing or curing hepatic steatosis after 12.5 months of follow-up when compared to SG^17^. This study used elastography as an accurate method to assess hepatic steatosis 2 months after bariatric surgery.

Lassailly et al.^13^ showed that most patients undergoing bariatric surgery have regression of NAHS, regardless of the technique used. We can conclude that these results are due to the significant weight loss which occurred 1-year after bariatric surgery, regardless of the use of SG or RYGB. Considering that the change in incretins may have an early effect on hepatic steatosis, we devised whether, in 2 months after bariatric surgery, there would be significant changes in fibrosis and hepatic steatosis or if the technique used would be important in this change.

Another objective of this study was to evaluate which of the surgical techniques performed would be able to reduce hepatic steatosis and fibrosis early and more intensely. Previous studies showed that the use of Fibroscan^®^ compares favorably with liver biopsy, even in obese patients^23^.

When the data are crossed comparing preoperative and postoperative steatosis and fibrosis in each surgical group, we can see that those who underwent RYGB had improvement in both fibrosis and hepatic steatosis within 2 months postoperatively. However, the same finding does not occur in the SG group. The incretin changes generated by RYGB are likely the mainstay of this improvement in hepatic steatosis and fibrosis, as it is also in glycemic control in patients with type 2 diabetes mellitus^21^.

Even with a small sample of patients, the results are compatible with previous studies, which showed an improvement in steatosis, more evident in patients undergoing RYGB. However, studies with larger casuistries are needed in the future to confirm these changes in the early postoperative period of bariatric surgeries.

## CONCLUSION

This study demonstrates that liver elastography is a suitable method with accuracy to demonstrate the reduction of hepatic fibrosis and steatosis in the early postoperative period of bariatric surgery. In addition, elastography showed that patients undergoing RYGB had a more significant steatosis reduction than SG.
